# Nicotine Bitartrate Reduces Falls and Freezing of Gait in Parkinson Disease: A Reanalysis

**DOI:** 10.3389/fneur.2019.00424

**Published:** 2019-05-07

**Authors:** Abraham Lieberman, Thurmon E. Lockhart, Markey C. Olson, Victoria A. Smith Hussain, Christopher W. Frames, Arshia Sadreddin, Margaret McCauley, Elizabeth Ludington

**Affiliations:** ^1^Muhammad Ali Parkinson Center, Barrow Neurological Institute, St. Joseph's Hospital and Medical Center, Phoenix, AZ, United States; ^2^Ira A. Fulton Schools of Engineering, Arizona State University, Phoenix, AZ, United States; ^3^California Pacific Neuroscience Institute, California Pacific Medical Center, San Francisco, CA, United States; ^4^Neuraltus Pharmaceuticals, Inc., San Bruno, CA, United States

**Keywords:** Dyskinesia, falls, freezing of gait, nicotine, Parkinson disease

## Abstract

**Objective:** Determine if NC001, an oral formulation of nicotine that reduces levodopa-induced dyskinesias (LIDs) in MPTP-Parkinson monkeys, could reduce falls, freezing of gait (FOG), and LIDs in Parkinson disease (PD) patients.

**Methods:** Previously collected data from a study analyzing the effects of NC001 on LIDs in PD patients were reanalyzed. Because indirect-acting cholinergic drugs are sometimes helpful in reducing falls, we hypothesized that NC001, a direct-acting cholinergic agonist, could reduce falls in PD. The original 12-center, double-blind, randomized trial enrolled 65 PD patients. NC001 or placebo was administered 4 times per day for 10 weeks, beginning at 4 mg/day and escalating to 24 mg/day. Assessments included the Unified Dyskinesia Rating Scale (UDysRS) and Parts II-III of the original Unified Parkinson's Disease Rating Scale (UPDRS).

**Results:** Randomization (1:1) resulted in 35 patients on NC001 and 30 on placebo at baseline. Thirty and 27 patients, respectively, had data available for an intent-to-treat analysis. NC001 was safe and well-tolerated. After 10 weeks, NC001 patients (14/30) had a significant reduction in falls vs. placebo patients (3/27) (*p* = 0.0041) as assessed by UPDRS Part II. NC001 patients (12/30) also had significantly reduced FOG vs. placebo patients (4/27) (*p* = 0.0043). NC001 patients, compared with placebo patients, had a significant improvement (*p* = 0.01) in UDysRS ambulation subtest (40% vs. 3%, respectively). Although NC001 patients had a greater reduction in dyskinesias on the UDysRS than placebo patients (30% vs. 19%, respectively), this was not significant (*p* = 0.09).

**Conclusions:** NC001 significantly improved two refractory symptoms of PD, falls and FOG. The reduction in falls and FOG is attributed to selective stimulation of nicotinic receptors.

**Clinical Trial Registration:** Conducted under IND 105, 268, serial number 0000. ClinicalTrials.gov identifier NCT00957918.

## Introduction

Parkinson disease (PD) is a common and disabling neurodegenerative disease. Although PD is not curable, many of its symptoms can be managed by dopaminergic drugs, principally levodopa. However, as PD progresses, more than 30% of patients develop freezing of gait (FOG), a sudden, transient, and often unpredictable inability to walk (1, 2). FOG is often disabling, leads to falls, and is relatively refractory to levodopa (1). Advancing PD also causes more than 30% of patients to fall (3–5). These falls may or may not be associated with FOG (1, 2). Falls are the main reason PD patients are hospitalized, leading to high health-care expenses (3, 5). Falls are often refractory to, and sometimes worsened by, levodopa (6). The dose of levodopa is generally increased as the disease progresses, which results in levodopa-induced dyskinesias (LIDs) in at least 30% of patients (7). LIDs interfere with activities of daily living, including ambulation.

NC001 (nicotine bitartrate dihydrate, originally named NP002), a novel, orally administered, direct agonist at central nervous system cholinergic nicotine receptors, was evaluated in PD. NC001, like nicotine in cigarettes, results in a rapid, high-level, selective stimulation of nicotine receptors and may be neuroprotective (8, 9). Unlike nicotine from cigarettes, NC001 is uncontaminated by potentially carcinogenic tars and fillers, and, unlike nicotine in patches and gums, NC001 does not desensitize nicotinic receptors (8–10). Nicotinic receptors are distributed throughout the central nervous system with clusters in the striatum and the pedunculopontine nuclei (PPN) (8–11). Before the initiation of this study, NC001 was found to reduce LIDs in monkeys with 1-methyl-4-phenyl-1, 2, 3, 6-tetrahydropyridine (MPTP)-induced lesions. Donepezil or rivastigmine, which are indirect agonists at muscarinic cholinergic receptors, did not have this effect (8, 10).

**Figure 1:**
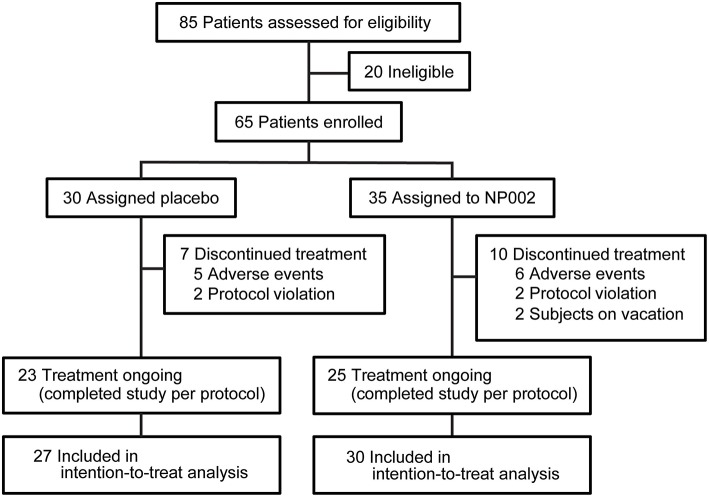
Trial profile. The number of patients screened and enrolled in the study, as well as the number of patients who left the study at each point and the reasons for exclusion from the study, with the resulting number left enrolled, are shown. All patients who completed at least one postbaseline visit were included in the intention-to-treat analysis, which is also documented in the figure. Used with permission from Barrow Neurological Institute, Phoenix, Arizona.

In 2009, a study involving 65 patients from 12 centers was undertaken to evaluate the safety and tolerability of NC001 in patients with PD, and the effectiveness of NC001 on LIDs, as assessed by the Unified Dyskinesia Rating Scale (UDysRS) (12), and on rigidity, tremor, and bradykinesia as assessed by the Unified Parkinson's Disease Rating Scale (UPDRS) (ClinicalTrials.gov identifier: NCT00957918) (Figure 1) (13). The results suggested a modest but nonsignificant reduction in LIDs and no improvement in rigidity, tremor, or bradykinesia, symptoms that are usually improved by levodopa. Although the scales utilized for the original study contain questions pertaining to falls and FOG, we did not initially analyze these data.

Falls and FOG are symptoms usually not improved by levodopa (5, 6), leading investigators to study the effects of other neurotransmitters. Bohnen et al. (14) and Perez-Lloret and Barrantes (15) have studied the effect of the cholinergic system on locomotion, postural control, and falls, while Chung et al. (16) studied the effects of donepezil on reducing falls and Henderson et al. (17) studied the effects of rivastigmine in improving postural control and possibly in reducing falls (but not FOG). On the basis of our observations of the potential benefits of these cholinergic drugs in reducing falls, we analyzed our previously collected data on the effects of orally administered NC001 vs. a placebo, specifically focusing on falls and FOG in patients with PD. Because nicotine directly stimulates nicotinic cholinergic receptors in the central nervous system, we hypothesized that NC001 would reduce falls and FOG in PD patients.

## Materials and Methods

### Study Design

The original study was a randomized, double-blind, multicenter study developed to compare NC001 with placebo in adult PD patients. The study was completed in 12 centers within the United States. Both private practices and university hospitals participated in data collection. The study was conducted in accordance with the Declaration of Helsinki and Good Clinical Practice Guidelines. All sites received approval from an institutional review board. The clinical trial was conducted under IND 105, 268, ClinicalTrials.gov identifier NCT00957918. This paper is a result of a retrospective reanalysis of our data, based on data from new studies on the effects of indirect-acting cholinergic agents on falls and FOG.

### Patients

Eligible study patients were required to have a diagnosis of idiopathic PD, to be within a Hoehn and Yahr stage II–IV (13) while in a peak “on” state (levodopa level in a therapeutic range), to have been on a stable dose of levodopa for at least 30 days prior to and throughout the study, to have moderate to severely disabling LIDs for at least 25% of the waking day as determined by Questions 32 and 33 in Part IV of the UPDRS (13), and to have a Mini-Mental State Examination score ≥26. Patients were excluded from the study if they were active smokers or had atypical Parkinson disorders; prior deep brain stimulation (DBS); unstable angina; or a history of ventricular arrhythmias, active peptic ulcer, schizophrenia, schizoaffective disorder, or bipolar disease.

Patients were recruited from the practices of the participating study investigators. There were no advertisements or monetary inducements to patients. Written informed consent was obtained from each patient prior to participation.

### Randomization and Masking

PD patients were randomly assigned to either the NC001 or placebo group in a 1:1 ratio. Random assignment was based on a computer-generated process, and investigational product was distributed based on a randomized number written on the investigational product container, so as to avoid unblinding.

The NC001 and placebo were identical in appearance and packaging. Both nicotine bitartrate dehydrate and placebo were manufactured by Siegfried Ltd. (Zofingen, Switzerland) and were packaged, tested for clinical use, and monitored for stability in accordance with current Good Manufacturing Practices by UPM Pharmaceuticals (Baltimore, Maryland, USA). All patients, study site personnel, raters, and the sponsor were blinded to treatment assignment. Active drug and placebo were identical in shape, color, weight, appearance, and dissolvability. Packaging was identical.

### Procedures

The duration of the study was 17 weeks, including 10 weeks of active treatment with NC001 or placebo, a transition period (3 weeks), and a posttreatment period (4 weeks). The drug or placebo was self-administered orally 4 times daily in a blinded fashion. Patients were examined at each visit as described below (see *Outcomes*) while in an “on” state, when levodopa was in a therapeutic range. During the treatment phase, dosing was begun at 1 mg every 6 h (total daily dose of 4 mg/day) at the time of the baseline visit and escalated upward at 2-week intervals as follows: 2 mg every 6 h at visit one (8 mg/day); 4 mg every 6 h at visit two (16 mg/day); and 6 mg every 6 h at visit three (24 mg/day). Patients were maintained on 24 mg/day for 4 weeks.

### Outcomes

The primary outcomes in the original study were changes from baseline to week 10 in UPDRS and UDysRS scores by treatment group. In our reanalysis, questions from the original UPDRS Part II (Activities of Daily Living, Questions 13 and 14) and Part III (Motor Examination, Item 30, the “pull test,” a test of postural stability) were compared to assess for treatment effects on falls and FOG after administration of NC001 or placebo. LIDs before and after treatment were assessed with the UDysRS (12) during both analyses. Evaluations during transition and posttreatment periods were identical to those during the treatment phase.

Compliance was checked with serum cotinine levels (cotinine is a metabolite of nicotine). Nicotine withdrawal symptoms were treated after completion of the active trial and again after completion of the posttreatment period utilizing the Nicotine Withdrawal Symptom Assessment (18). Adverse events potentially related to treatment were reported by frequency.

### Statistical Analysis

All hypotheses were tested using 2-sided tests with alpha set at the 0.05 level of significance. In general, efficacy data were summarized by treatment group, and safety data were summarized by treatment group and for the overall study population. In all analyses, assumptions such as normality and homogeneity of variance were examined before conducting the proposed parametric statistical procedures. Categorical variables were analyzed by Fisher exact 2-tailed tests and continuous variables were tested under 2-sample *t*-tests. The equality of variances was examined using an F-test before applying the 2-sample *t*-test. The *t*-test statistics were adjusted if the variances between groups were significantly unequal.

The modified intent-to-treat population comprised all patients who were in the randomized population, took at least 1 dose of the study medication, and had a baseline and at least 1 scheduled postbaseline assessment. The efficacy analyses were conducted using this population. The intent-to-treat population consisted of all subjects who were in the randomized population who took at least 1 dose of study medication. This population was used only for the *post hoc* sensitivity analysis.

Sample size calculations were based on the following assumptions: type I error of α = 0.05, power = 70%, 1-sided test, placebo response rate = 36.8%, and a 30% improvement in response compared to placebo. Initial protocol power calculations were based on a standard formula for power in 2 × 2 tables using Stata v10.1. The calculations were confirmed by using a 2-group chi-square test of equal proportions using N-Query Advisor 6.01. The calculations showed that, when using a 1-sided test, 25 evaluable subjects per arm would provide a 70% chance of detecting a difference between placebo and NC001.

For falls (UPDRS Part II, Question 13), a 5-point scale was used: 0 = no falls to 4 = falls more than once per day. For FOG and falls related to FOG (UPDRS Part II, Question 14), a 5-point scale was used: 0 = no FOG to 4 = frequent falls from FOG. Retropulsion, which assesses postural control by the pull test (UPDRS Part III, Item 30) on a 5-point scale from 0 to 4, was also compared between NC001 and placebo patients. A comparison of distributions between the NC001 and placebo groups was made using a distribution that ranged from−3 (a 3-point improvement on the 5-point scale) to +3 (a 3-point worsening on the 5-point scale). Analyses were made with the Fisher exact 2-tailed test. Improvement or worsening over baseline was calculated from the last treatment visit (19).

The efficacy variables for LIDs were the mean change from baseline to week 10 in the UDysRS total score and UDysRS subscores. An analysis of covariance combined features of regression and analysis of variance. Exploratory analyses of continuous outcomes using a mixed-effect model repeated measures method were performed on the modified intent-to-treat population in conjunction with the analysis of covariance model to assess the sensitivity of the data to different analysis methods. *Post hoc* sensitivity analyses were done on the intent-to-treat population using both the analysis of covariance and mixed-effect model repeated measures methods. In addition to the descriptive summary of each variable from baseline to end point, least-squares (LS) means, standard errors, and 95% confidence intervals (CI) of each group, and the difference of LS means between the 2 treatment groups and its 95% CI are presented. A similar analysis was applied to comparisons between UPDRS Part II and UPDRS Part III.

The original study was overseen by a data monitoring company, i3, a contract research organization located in Basking Ridge, New Jersey, USA.

### Role of the Funding Source

The sponsor of the study had no role in study design, data collection, data analysis, data interpretation, or writing of the report. The corresponding author had full access to all the data in the study and had final responsibility for the decision to submit.

## Results

Study recruitment proceeded from September 30, 2009, to September 19, 2010. Eighty-five participants were assessed for eligibility, and 20 were determined to be ineligible for the study. Sixty-five patients were randomized: 35 to NC001 and 30 to placebo. For falls, FOG, and retropulsion, 30 NC001 and 27 placebo patients had sufficient data to be analyzed. Over the course of the study, 10 patients assigned to the NC001 group and 7 assigned to the placebo group dropped out of the study. All patients who participated in at least 1 postbaseline visit were included in the statistical analysis, 30 NC001 patients and 27 placebo patients. The demographics of all 65 patients recruited at baseline are summarized in Table 1.

**Table 1:** Baseline characteristics of the modified intent-to-treat population.

	**NC001 (*n* = 35)**	**Placebo (*n* = 30)**	***P*-Value**
Sex			0.22
Male	14 (40.0%)	17 (56.7%)	
Female	21 (60.0%)	13 (43.3%)	
Age (years)	68.1 ± 8.3	65.5 ± 7.2	0.18
Ethnic origin			0.78
White	33 (94.3%)	28 (93.3%)	
Black	1 (2.9%)	0 (0.0%)	
Asian	1 (2.9%)	2 (6.7%)	
PD duration (years)	11.2 ± 4.7	11.1 ± 5.6	0.93
Levodopa duration (years)	9.6 ± 4.7	10.2 ± 5.4	0.66
LIDs duration (years)	5.3 ± 3.3	5.2 ± 3.2	0.97
Levodopa dose (mg/day)	612 ± 201	582 ± 180	0.70
Hoehn and Yahr stage	2.52 ± 0.57	2.38 ± 0.48	0.97

*Data are n (%) or least-squares means ± SE. PD, Parkinson disease; LIDs, levodopa-induced dyskinesias. P-values for NC001 vs. placebo groups calculated using 2-sample t tests*.

The changes in UPDRS Part II and III scores from baseline to 10 weeks (i.e., posttreatment) were compared between NC001 and placebo patients. No significant differences were found after treatment between the NC001 and placebo groups for either Part II or Part III (*p* ≥ 0.34) (Table 2).

**Table 2:** Comparison by treatment group of mean UPDRS Part II and Part III scores before and after treatment.

	**UPDRS II**		**UPDRS III**	
	**(Activities of daily living)**		**(Motor examination)**	
	**NC001**	**Placebo**	**NC001**	**Placebo**
LS means ± SE^*^				
Baseline	13.1 ± 6.0	11.4 ± 5.3	20.0 ± 8.8	16.9 ± 8.3
Week 10	10.1 ± 5.3	8.8 ± 5.0	17.3 ± 10.6	16.8 ± 8.6
Change	2.8 ± 5.2	2.7 ± 4.5	2.0 ± 7.5	−0.4 ± 6.5
NC001 vs. placebo^†^				
Difference in LS means ± SE (95% CI)	10.1 ± 0.96 (6.9–12.1)		−18.3 ± 1.8 (−24.1 to −16.3)	
*P*-value	0.63		0.34	

* At baseline, n = 35 NC001 patients and n = 30 placebo patients. At 10 weeks, n = 30 NC001 patients and n = 27 placebo patients.

† Comparison of baseline to week 10. LS, least squares; UPDRS, Unified Parkinson's Disease Rating Scale.

Scores by treatment group for UPDRS Part II Questions 13 and 14 and Part III Item 30, the pull test, were compared separately (Table 3). Fourteen of 30 (47%) NC001 patients had a reduction in falls. In contrast, only 3 of 27 (11%) placebo patients had a reduction in falls. This difference was statistically significant (*p* = 0.0043). The difference in reduction of FOG was also significant (*p* = 0.0041) between groups, with 12 of 30 (40%) NC001 patients and 4 of 27 (15%) placebo patients having a reduction in FOG. Similarly, 10 of 30 (33%) NC001 patients had a reduction in retropulsion, the pull test, compared to only 2 of 27 (7%) placebo patients (*p* = 0.02).

**Table 3:** Change by treatment group in specific UPDRS scores before and after treatment.^*^

		**Change in score: baseline to week 10**						
		**(no. of patients)**						
	**Improvement**	**-3**	**-2**	**-1**	**No change**	**+1**	**+2**	**+3**
Falls								
NC001	14/30 (47%)	2	5	7	15	1	0	0
Placebo	3/27 (11%)	0	0	3	23	1	0	0
*P*-value^†^	0.0041							
FOG								
NC001	12/30 (40%)	0	2	10	17	1	0	0
Placebo	4/27 (15%)	0	1	3	20	2	1	0
*P*-value^†^	0.0043							
Pull Test								
NC001	10/30 (33%)	0	5	5	19	1	0	0
Placebo	2/27 (7%)	0	0	2	24	1	0	0
*P*-value^†^	0.02							

* UPDRS Part II Question 13 assesses falls unrelated to freezing of gait (FOG), Part II Question 14 assesses FOG, and Part III Item 30 assesses retropulsion (pull test). Improvement is represented as a - (negative) or decrease in falls or FOG. The number of patients included everyone who had a baseline and at least 1 postbaseline visit.

† Change in NC001 and placebo patient groups scores were compared using a Fisher exact 2-tailed test. UPDRS, Unified Parkinson's Disease Rating Scale.

On the UDysRS ambulation subtest score, NC001 patients had a 46% reduction (i.e., improvement) in the effect of LIDs on ambulation (9.5 ± 6.0 at baseline to 5.1 ± 5.3 at week 10) while placebo patients had a 3% reduction (7.6 ± 5.0 at baseline to 7.4 ± 5.7 at week 10). This difference was significant in favor of NC001 (*p* = 0.01). On the total UDysRS, the main measure of LIDs change, there was a 29% reduction in LIDs among patients receiving NC001 (51.7 ± 16.9 at baseline to 36.4 ± 16.1 at week 10) and a 19% reduction among patients on placebo (48.1 ± 15.0 at baseline to 38.8 ± 19.0 at week 10). Although the percentage reduction in total UDysRS favored NC001, the difference between groups did not reach statistical significance (*p* = 0.09) (Table 4).

**Table 4:** Comparison of LS mean ± SE Unified Dyskinesia Rating Scale (UDysRS) scores before and after treatment by treatment group.

	**NC001 patients**		**Placebo patients**		**Difference in LS means at week 10**		
	**Baseline (*n* = 30)**	**Week 10 (*n* = 27)**	**Baseline (*n* = 27)**	**Week 10 (*n* = 24)**		**95% CI**	***P*-value**
Evaluation of dyskinesias on:							
7 body parts	13.5 ± 5.4	8.5 ± 5.4	12.7 ± 4.9	10.3 ± 6.1	−2.8 ± 2.6	−5.7 to 0.1	0.06
Communication	10.5 ± 4.8	7.0 ± 5.0	9.4 ± 5.2	8.2 ± 5.7	−1.9 ± 1.4	−4.7 to 0.9	0.19
Drinking	9.1 ± 5.6	5.3 ± 5.0	7.8 ± 5.4	7.0 ± 5.1	−2.4 ± 1.7	−5.8 to 1.0	0.17
Dressing	11.3 ± 6.2	6.9 ± 5.5	11.1 ± 5.1	8.7 ± 5.4	−2.1 ± 1.4	−5.0 to 0.7	0.14
Ambulation	9.5 ± 6.0	5.1 ± 5.3	7.6 ± 5.0	7.4 ± 5.7	−3.7 ± 1.9	−6.5 to −0.9	**0.01**
Total UDysRS score	51.7 ± 16.9	36.4 ± 16.1	48.1 ± 15.0	38.8 ± 19.0	−6.7 ± 4.1	−14.7 to −1.3	0.09

*Part 1A UDysRS is a patient assessment of duration and severity of dyskinesia. This was replaced by UPDRS Part IV Questions 32-33. Part 1B UDysRS is a patient assessment of the impact of dyskinesia on ADLs. This was replaced by the Lang-Fahn ADL Dyskinesia Scale. Part 2A UDysRS assesses time in “off”-dystonia. This was not included in the UDysRS total. Part 2B UDysRS assesses impact of “off” dystonia. No significant differences were noted after 10 weeks of treatment from baseline between NC001 and placebo. Part 2B was not included in the UDysRS total. Part 3 UDysRS is a physician assessment of impact of dyskinesia on 7 body parts: face, neck, both arms and legs, and trunk and a physician assessment of the impact of dyskinesia on communication, drinking from a cup, dressing, and ambulation. Bold value indicates statistical significance. LS, least squares*.

No nicotine withdrawal symptoms were noted for patients on NC001 or placebo. Serum cotinine levels paralleled dosing of nicotine in NC001 patients and were absent in placebo patients. The most frequently seen adverse events are reported in Table 5. The most common adverse event among NC001 patients was nausea (13/35, 37.1%). This generally resolved spontaneously or with ondansetron. The most common adverse event among placebo patients was insomnia (3/30, 10.0%). More adverse events (45 events) were reported among NC001 than placebo patients (14 events). Eleven patients withdrew because of adverse events, 6 who were on NC001 and 5 who were on placebo.

**Table 5:** Adverse events (AEs) among patients receiving NC001 or placebo.

**AE**	**No. (%) of patients**	
	**NC001 (*****N*** **=** **35)**	**Placebo (*****N*** **=** **30)**
Any AE	26 (74.3)	14 (46.7)
Nausea	13 (37.1)	2 (6.7)
Dizziness	7 (20.0)	1 (3.3)
Constipation	5 (14.3)	1 (3.3)
Vomiting	4 (11.4)	0 (0.0)
Fatigue	3 (8.6)	0 (0.0)
Pain	3 (8.6)	0 (0.0)
Diarrhea	2 (5.7)	1 (3.3)
Headache	2 (5.7)	1 (3.3)
Pain in extremity	2 (5.7)	0 (0.0)
Tremor	2 (5.7)	0 (0.0)
Nightmare	2 (5.7)	0 (0.0)
Insomnia	0 (0.0)	3 (10.0)

*Eleven patients withdrew because of adverse effects: 6 NC001 patients, 5 placebo patients*.

## Discussion

The study demonstrated that NC001 administered for 10 weeks, when compared to placebo, significantly reduced falls (*p* = 0.0043) and FOG (*p* = 0.0041) independently in PD patients. Falls and FOG are among the most debilitating consequences of PD (2–5). No current treatment has been shown to consistently reduce both falls and FOG. Chung et al. (16) demonstrated a reduction in falls using donepezil. They enrolled 23 patients who reported falling at least twice per week in a double-blind crossover trial. Fall frequency on placebo was 0.25 ± 0.08 per day vs. 0.13 ± 0.03 for patients on donepezil (*p* < 0.05). Henderson et al. (17) demonstrated an improvement in locomotion (dynamic stability) but not a reduction in falls in patients on rivastigmine. Neither of these studies mentioned FOG. Donepezil and rivastigmine are indirect cholinomimetics, releasing acetyl choline at muscarinic receptors. Antimuscarinic drugs increase FOG (9, 15), but it is unknown whether muscarinic drugs reduce FOG. NC001 is a direct agonist at nicotinic receptors. This selectivity may account for the beneficial effects of NC001 on both falls and FOG.

Cholinergic activity is reduced in PD, and this reduction appears to be closely linked to falls (14–17). Perez-Lloret and Barrantes (15) studied 17 fallers and 27 non-fallers with PD with PET scans utilizing [^11^C] methyl-4-piperidinyl propionate acetyl-cholinesterase (a marker for cholinergic activity) and [^11^C] dihydro-tetrabenazine vesicular monoamine transporter type (a marker for dopaminergic activity). Cholinergic activity was lower only in fallers. Dopaminergic activity did not change between fallers and non-fallers, which implied that falls are mainly caused by reduced cholinergic activity. NC001 reduced falls and FOG, we believe, through a direct effect on nicotinic receptors on cholinergic neurons (9, 15, 20–23).

It is hypothesized that this effect was primarily active within the PPN. The PPN has rich connections to the substantia nigra reticulata, the subthalamic nucleus, and the primary motor and premotor cortices (9, 16, 21, 24) and is affected in PD, showing a progressive loss of cholinergic neurons (21–23). Karachi et al. (25) demonstrated that, in primates with an intact dopaminergic system, bilateral lesions of the PPN resulted in marked deficits in locomotion and postural control. Bensaid et al. (23) demonstrated in primates that cholinergic and dopaminergic interactions, including those in the PPN, are important and suggested that, although each transmitter has different functions, both are needed for postural control and locomotion (20). Human studies have also linked loss of PPN cholinergic neurons to disability in PD (9, 21, 22, 24). The PPN is pivotal in scaling movement to proprioceptive and vestibular inputs and may be pivotal in reducing falls and FOG (22). The literature suggests that medically refractory falls and FOG can improve with DBS of the PPN (26).

NC001 also may have induced a reduction in falls and FOG by reducing LIDs. Although we believe the reduction is related to an improvement in postural control, as demonstrated by improvement in the pull test, further study is needed to demonstrate this mechanism. A study in which patients experiencing falls and/or FOG are quantitatively studied during standing, gait initiation, and locomotion could resolve this question. Such quantitative data can be collected with inertial measurement units, kinematic motion capture systems, force plates, and surface electromyography. Whatever the mechanism, effectiveness in reducing falls and FOG would be a major step forward in the treatment of PD.

The effect of NC001 on LIDs was mixed. NC001 decreased the UDysRS total score by 29% vs. 19% for those on placebo and significantly decreased the scores regarding the effect of LIDs on ambulation (*p* = 0.01). The effect of NC001 on LIDs was not as robust as the effect reported for extended-release amantadine (27). However, amantadine was not shown, in a double-blind study, to reduce falls or FOG. Given the debilitating effects of LIDs in many patients, the fact that amantadine is the only drug available to treat LIDs, and that amantadine does not reduce LIDs in all patients, there appears to be a role for NC001 in treating LIDs.

It is important to note the limitations of the results of this study. Because this study was a retrospective reanalysis of previously collected data, which focused on LIDs instead of falls or FOG, subjects were not randomized on the basis of falls or FOG. At the time of the original study, best practices were self-reporting of falls and FOG. In retrospect, we find that there are limitations to such self-reporting. This study provides only exploratory, proof-of-concept data, and a future study to analyze actual changes in falls and FOG while patients are on NC001 is needed. The short duration of the study (10 weeks) is a limitation, as this time period may be insufficient to demonstrate long-term reductions in falls and FOG. Future studies will rely on force plates, motion capture systems, and inertial measurement units that can record the quantitative features of balance and gait parameters, FOG, and falls in real time.

## Author Contributions

AL supervised the study, participated in the reanalysis, evaluated patients in the original study. TL, MO, VS, CF, and MM reanalysis and interpretation. AS evaluated patients in the original study. EL, statistical analysis.

### Conflict of Interest Statement

Neuraltus Pharmaceuticals, Inc., funded the original study but did not fund the reanalysis of the data. EL is employed by Neuraltus Pharmaceuticals, Inc. The remaining authors declare that the research was conducted in the absence of any commercial or financial relationships that could be construed as a potential conflict of interest.

## Abbreviations

CI: confidence interval
DBS: deep-brain stimulation
FOG: freezing of gait
LID: levodopa-induced dyskinesia
LS: least squares
PD: Parkinson disease
PPN: pedunculopontine nuclei
UDysRS: Unified Dyskinesia Rating Scale
UPDRS: Unified Parkinson's Disease Rating Scale.
